# Evolution of the core and pan-genome of *Streptococcus*: positive selection, recombination, and genome composition

**DOI:** 10.1186/gb-2007-8-5-r71

**Published:** 2007-05-02

**Authors:** Tristan Lefébure, Michael J Stanhope

**Affiliations:** 1 Department of Population Medicine and Diagnostic Sciences, College of Veterinary Medicine, Cornell University, Ithaca, NY 14853, USA

## Abstract

**Background:**

The genus *Streptococcus *is one of the most diverse and important human and agricultural pathogens. This study employs comparative evolutionary analyses of 26 *Streptococcus *genomes to yield an improved understanding of the relative roles of recombination and positive selection in pathogen adaptation to their hosts.

**Results:**

*Streptococcus *genomes exhibit extreme levels of evolutionary plasticity, with high levels of gene gain and loss during species and strain evolution. *S. agalactiae *has a large pan-genome, with little recombination in its core-genome, while *S. pyogenes *has a smaller pan-genome and much more recombination of its core-genome, perhaps reflecting the greater habitat, and gene pool, diversity for *S. agalactiae *compared to *S. pyogenes*. Core-genome recombination was evident in all lineages (18% to 37% of the core-genome judged to be recombinant), while positive selection was mainly observed during species differentiation (from 11% to 34% of the core-genome). Positive selection pressure was unevenly distributed across lineages and biochemical main role categories. *S. suis *was the lineage with the greatest level of positive selection pressure, the largest number of unique loci selected, and the largest amount of gene gain and loss.

**Conclusion:**

Recombination is an important evolutionary force in shaping *Streptococcus *genomes, not only in the acquisition of significant portions of the genome as lineage specific loci, but also in facilitating rapid evolution of the core-genome. Positive selection, although undoubtedly a slower process, has nonetheless played an important role in adaptation of the core-genome of different *Streptococcus *species to different hosts.

## Abstract 2

Comparative evolutionary analyses of 26 *Streptococcus *genomes show that recombination and positive selection have both had important roles in the adaptation of different species to different hosts.

## Background

Microbial pathogens show surprising capacity for adaptation to new hosts, antibiotics, or immune systems. Three principal mechanisms are regarded as important in this adaptive potential: Darwinian, or positive selection, favoring the fixation of advantageous mutations; acquisition of new genetic material by lateral DNA exchange (that is, recombination); and gene regulation. Several studies have suggested that recombination might be the key factor in adaptation of pathogens and that the recombination rates of bacteria might be higher than their mutation rates [1-4]. At the same time, there is a portion of the genome - the core-genome - that is thought to be representative of bacterial taxa, at various taxonomic levels [5]. Recent molecular evolution analyses of *Escherichia coli *and *Salmonella enterica *[6,7] have identified genes under positive selection pressure in the core-genome of these enteric bacteria. Genome sequence data are now available for numerous species of several genera of bacteria, providing the possibility of using comparative evolutionary genomic approaches to assess positive selection pressure and the role of horizontal gene transfer in the evolution of the core-genome of a bacterial genus.

One such important bacterial genus is *Streptococcus*, which includes some of the most important human and agricultural pathogens, causing a wide range of different diseases, and inflicting significant morbidity and mortality throughout the world, as well as resulting in significant economic burden. Twenty six genomes of *Streptococcus *are available on public databases belonging to six different species, including *S. pneumoniae*, *S. agalactiae*, *S. pyogenes*, *S. thermophilus*, *S. mutans *and *S. suis*. *S. pyogenes *(Group A *Streptococcus*; GAS), is responsible for a wide range of human diseases, including pharyngitis, impetigo, puerperal sepsis, necrotizing fasciitis ('flesh-eating disease'), scarlet fever, the postinfection sequelae glomerulonephritis and rheumatic fever. In addition, *S. pyogenes *has recently been associated with Tourette's syndrome and movement and attention deficit disorders [8]. A resurgence of *S. pyogenes *infections has been observed since the mid-1980s. *S. agalactiae *is another important human pathogen and is the leading cause of bacterial sepsis, pneumonia, and meningitis in US and European neonates [9]. Although *S. agalactiae *normally behaves as a commensal organism that colonizes the genital or gastrointestinal tract of healthy adults, it can cause life threatening invasive infection in susceptible hosts, such as newborns, pregnant women, and nonpregnant adults with chronic illnesses [10]. *S. agalactiae *was first recognized as a pathogen in bovine mastitis [11]. *S. pneumoniae *is the leading cause of human bacterial infection worldwide [12], although paradoxically, is primarily carried asymptomatically. It has been an object of medical study and scrutiny for over a century. *S. mutans *is implicated as the principal causative agent of human dental caries (tooth decay) [13]. *S. thermophilus *is a non-pathogenic, food microorganism, widely used in the dairy product industry. *S. suis *is responsible for a variety of diseases in pigs, including meningitis, septicemia, arthritis, and pneumonia [14]. It is also a zoonotic pathogen that causes occasional cases of meningitis and sepsis in humans, but has recently also been implicated in outbreaks of streptococcal toxic shock syndrome [15].

A recent comparative genomic analysis of five of these above mentioned streptococcal species (*S. suis *not included), focused on understanding the role of lateral gene transfer in shaping the genomes of each of these lineages, and analyzed some of the species specific genes for potential adaptive evolution [16]. Species or strain specific loci are often the focus of attempts to understand adaptive differences in bacteria. However, with the exception of the Chen *et al*. [7] study on *E. coli*, assessments of adaptive evolution in the core-genome components of other bacterial species have not been thoroughly explored. In addition to individual genome sequences for several species of *Streptococcus*, there are also complete genome sequences available for multiple strains of *S. agalactiae*, *S. pyogenes*, and *S. thermophilus*. Genome wide molecular selection analyses, designed to assess selection pressure across the entire core-genome of different species and strains of *Streptococcus *have not been reported, and also no published reports have attempted to address the relative role of selection versus recombination in the diversification of the core-genome of *Streptococcus*.

Along with the burgeoning increase in microbial genome sequence data there has been a concomitant development of sophisticated methods for detecting positive selection in protein coding genes. These methods can be used to compare orthologous DNA sequence data across the entire genomes of the available species within the genus *Streptococcus*. Ziheng Yang, Rasmus Nielsen and colleagues [17-21] have developed powerful statistical methods for detecting adaptive molecular evolution. Their methods compare synonymous and nonsynonymous substitution rates in protein coding genes and regard a nonsynonymous rate elevated above the synonymous rate as evidence for positive or Darwinian selection. Positive natural selection leads to the fixation of advantageous mutations driven by natural selection, and is the fundamental process behind adaptive changes in genes and genomes, leading to evolutionary innovations and species differences. A significant advancement on many earlier methods, which averaged over sites and time, their methods are designed to detect positive selection at individual sites and lineages [20]. Our study employs these powerful selection methods to assess positive selection pressure across the core-genome components of the genus *Streptococcus*, as well as several species of *Streptococcus*, while concomitantly assessing levels of recombination within the core-genome.

Concomitant with the identification of bacterial core-genomes, it has become evident that there is an apparently dispensable portion of bacterial genomes, consisting of partially shared and strain-specific genes that can, even within a particular species, represent a surprisingly large proportion (for example, [22]). The concept of dispensable portions of genomes implies that genes have been lost and gained since separation from common ancestors, which in turn implies that this loss and gain can be estimated from reconstructed genome composition. This sort of approach has been undertaken previously, including for a few species of *Streptococcus *[23], with one of the resulting conclusions being that gene gain tends to be much greater than gene loss. An additional purpose of this paper is to compare gene gain and loss within and between *Streptococcus *species, making use of the larger comparative data set of species and strains now available, and to compare that history with histories of positive selection and recombination in the core-genome.

## Results

### Pan-genome, core-genome, and evolution of genome composition

**Figure 1:**
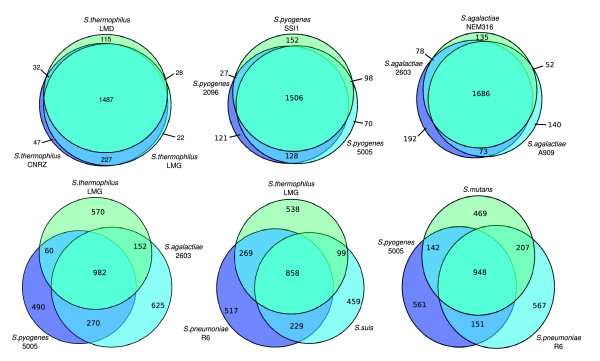
Venn diagram for six sets of three taxa. Above are taxa of the same species and below are taxa of different species. The surfaces are approximately proportional to the number of genes.

**Figure 2:**
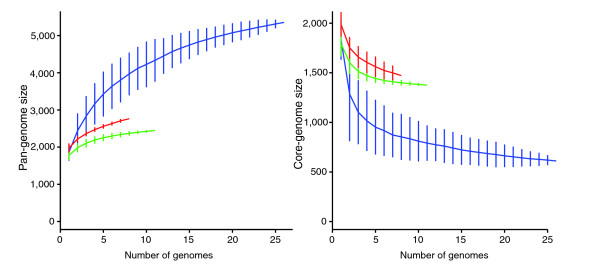
Accumulation curves for the total number of genes (left) or the number of genes in common (right) given a number of genomes analyzed for the different species of *Streptococcus *(in blue), the different strains of *S. agalactiae *(in red) and *S. pyogenes *(in green). The vertical bars correspond to standard deviations after repeating one hundred random input orders of the genomes.

**Figure 3:**
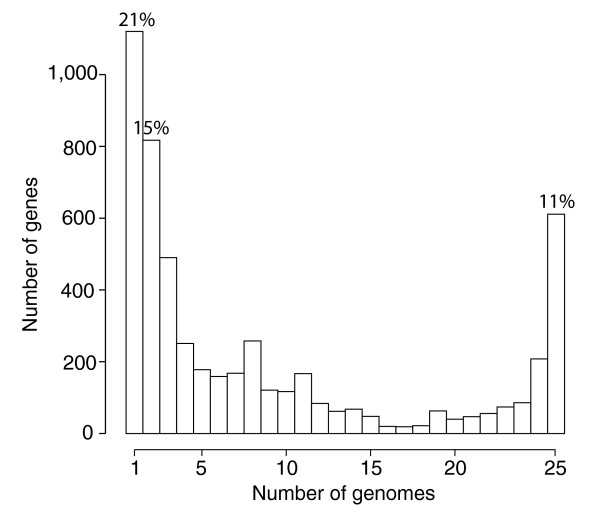
Frequency of genes within the 26 genomes included in this analysis. Genes present in a single genome represent lineage specific genes, while at the opposite end of the scale, genes found in all 26 genomes represent the *Streptoccocus *core-genome.

The number of protein coding genes per genome within the various strains and species of *Streptococcus *is relatively similar (ranging from 1,697 to 2,376; Table 1), but the gene composition of these genomes is much more variable. Based on the gene content table obtained by OrthoMCL (Additional data file 1), three strains of *S. agalactiae*, *S. pyogenes *or *S. thermophilus *share about 75% of their genes, and three different species of *Streptococcus *share only around half of their genes (Figure 1). This latter result appears to be independent of the particular strains or species involved in the comparison and of their phylogenetic affinities. Even with the inclusion of 26 genomes, the total number of possible genes - the pan-genome - of *Streptococcus *appears not to have been reached, as depicted in the gene accumulation curve (Figure 2), and we estimate the *Streptococcus *pan-genome probably surpasses 6,000 genes. A surprising 21% of the genes in the pan-genome of the genus *Streptococcus *(based on these 26 genome sequences), were represented in only one lineage, suggesting a remarkable degree of lateral gene transfer in shaping the genomes of each of these taxa (Figure 3). Within species, the pan-genome size also remains uncertain, although our estimates suggest that the pan-genome size of *S. pyogenes *is smaller, and better estimated with the currently available data, than that of *S. agalactiae *(Figure 2).

**Table 1:** Genomes analyzed

Species	Strain	Refseq accession number	Status	CDS	Serotype	References
*S. pyogenes*	MGAS10270	GenBank:NC_008022	Complete	1,987	M2	[46]
*S. pyogenes*	MGAS10750	GenBank:NC_008024	Complete	1,979	M4	[46]
*S. pyogenes*	MGAS2096	GenBank:NC_008023	Complete	1,898	M12	[46]
*S. pyogenes*	MGAS9429	GenBank:NC_008021	Complete	1,877	M12	[46]
*S. pyogenes*	M1 GAS	GenBank:NC_002737	Complete	1,697	M1	[76]
*S. pyogenes*	MGAS5005	GenBank:NC_007297	Complete	1,865	M1	[77]
*S. pyogenes*	MGAS8232	GenBank:NC_003485	Complete	1,845	M18	[78,79]
*S. pyogenes*	MGAS6180	GenBank:NC_007296	Complete	1,894	M28	[80]
*S. pyogenes*	MGAS315	GenBank:NC_004070	Complete	1,865	M3	[79]
*S. pyogenes*	SSI-1	GenBank:NC_004606	Complete	1,861	M3	[81]
*S. pyogenes*	MGAS10394	GenBank:NC_006086	Complete	1,886	M6	[82]
*S. pneumoniae*	R6	GenBank:NC_003098	Complete	2,043		[83]
*S. pneumoniae*	TIGR4	GenBank:NC_003028	Complete	2,094		[84]
*S. mutans*	UA159	GenBank:NC_004350	Complete	1,960		[85]
*S. agalactiae*	2603V/R	GenBank:NC_004116	Complete	2,124		[86]
*S. agalactiae*	A909	GenBank:NC_007432	Complete	1,996		[22]
*S. agalactiae*	NEM316	GenBank:NC_004368	Complete	2,094		[9]
*S. agalactiae*	515	GenBank:NZ_AAJP00000000	WGS	2,275		[22]
*S. agalactiae*	CJB111	GenBank:NZ_AAJQ00000000	WGS	2,197		[22]
*S. agalactiae*	COH1	GenBank:NZ_AAJR00000000	WGS	2,376		[22]
*S. agalactiae*	H36B	GenBank:NZ_AAJS00000000	WGS	2,376		[22]
*S. agalactiae*	18RS21	GenBank:NZ_AAJO00000000	WGS	2,146		[22]
*S. suis*	89/1591	GenBank:NZ_AAFA00000000	WGS	1,896		
*S. thermophilus*	CNRZ1066	GenBank:NC_006449	Complete	1,915		[87]
*S. thermophilus*	LMG 18311	GenBank:NC_006448	Complete	1,889		[87]
*S. thermophilus*	LMD-9	GenBank:NZ_AAGS00000000	WGS	1,835		

CDS, number of protein coding sequences; WGS, whole genome shotgun.

In contrast to the pan-genome estimates, the number of genes in common between the different species within the genus *Streptococcus *- the core-genome - appears to reach a plateau around 600 genes (Figures 2 and 3). Next to the genome specific genes and the genes shared by only two genomes, the genes of the core-genome were the third most common genes (11%; Figure 3), suggesting they form a coherent group. Similarly, the estimated core-genome for *S. pyogenes*, based on the 11 available strains, plateaus around 1,400 genes. The pattern was less clear for *S. agalactiae*, where the estimate of core-genome size does not level out, and appears as though it might still be influenced by the inclusion of new genome sequences. On the whole, these analyses suggest that it is possible to delineate a core-genome at both genus and species level. We analyzed four such core-genome data sets: the *Streptococcus *core-genome (611 genes), and the core-genomes of *S. agalactiae *(1,472 genes), *S. pyogenes *(1,376 genes) and *S. thermophilus *(1,487 genes). To save computation time, the *Streptococcus *core-genome data set was reduced to ten taxa by keeping only two strains per species for *S. agalactiae*, *S. pyogenes*, *S. thermophilus *(strains A909 and NEM316, MGAS9429 and M1 GAS, and CNRZ1066 and LMG 18311, respectively). After discarding clusters of genes containing paralogs (that is, clusters containing more than one gene per taxon), and alignments with uncertain site homologies, we obtained four data sets containing 260, 1,297, 1,212 and 1,365 genes representing the alignable core-genomes of *Streptococcus*, *S. pyogenes*, *S. agalactiae*, and *S. thermophilus*, respectively.

**Figure 4:**
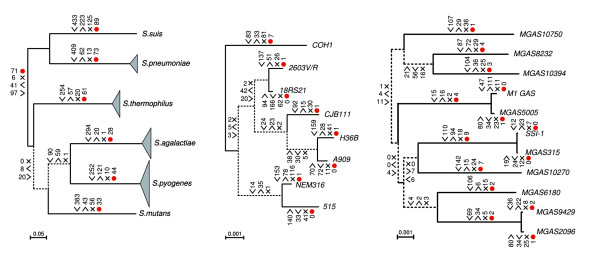
Gene gain, loss and duplication, and positive selection. Core-genome phylogenies of *Streptococcus *(left), *S. agalactiae *(middle), and *S. pyogenes *(right) based on concatenated genes. Dashed lines correspond to unresolved branches. Numbers adjacent to angle brackets facing the branch refer to genes gained, opposite direction - genes lost, and '×' refers to duplicated loci. Values correspond to the most parsimonious unambiguous changes, following an equally penalized model (that is, gain, loss and duplication events cost the same numbers of changes). Numbers adjacent to the red dot correspond to the number of genes under positive selection within the core-genome, on a particular lineage.

Determinations of the number of genes gained and lost on each of the lineages shows considerable variation (Figure 4) and, in agreement with earlier studies, gene gain was generally considerably greater than gene loss, as well as being particularly evident on external branches [23]. The lineage in the interspecific analysis showing the greatest gene gain was *S. suis*, followed closely by *S. pneumoniae *and *S. mutans*. Even within a species, between strains, the numbers of genes gained and lost were very high, reaching, for example, values in excess of 150 for gene gain in *S. agalactiae *strain H36B. High levels of gene gain and loss were evident, even for closely related isolates of the same serotype in *S. pyogenes *(for example, M1 GAS/MGAS5005; SSI-1/MGAS315; MGAS9429/MGAS2096). Branch lengths of the *S. pyogenes *concatenated tree were much longer than those for *S. agalactiae*, suggesting the lineages might be much older; however, despite this there was generally more gene gain on the *S. agalactiae *branches than on *S. pyogenes *branches. Large values for duplications were also a feature of the lineage specific evolution (Figure 4). Phylogenetic analysis of several of these cases suggests this is a combination of lineage specific duplications as well as LGT events involving homologous sequences from other species of *Streptococcus*. When gene gain was penalized with respect to gene loss (for example, [24]), not surprisingly, it globally decreased the number of gene gains and increased the number of gene losses (Additional data file 3) and, as a consequence, increased the number of genes in the pan-genomes of ancestral nodes (data not shown). Nevertheless, even with a penalty, gene gain remained in excess of gene loss on some lineages (Additional data file 3).

### Recombination

#### Between species of *Streptococcus*

**Figure 5:**
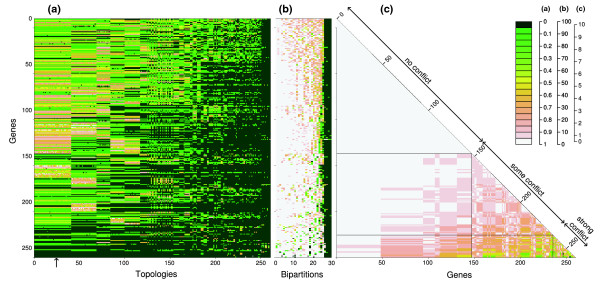
*Streptococcus *recombination heatmaps. Heatmaps of the (a) AU test, (b) bipartitions bootstrap scores and (c) well supported conflicting bipartitions on the core-genome of *Streptococcus*. Topologies are ordered from the less rejected (on the left) to the most rejected (on the right). Bipartitions are ordered from the less supported (on the left) to the most supported (on the right), and only bipartitions supported by at least a 70% bootstrap score are represented. Genes are ordered from the less conflicting (left and top) to the most conflicting (right and bottom). The well supported conflicting bipartitions heatmap represents a symmetrical distances matrix, where each cell corresponds to the number of well supported (that is, bootstrap ≥90) conflicting bipartitions between two genes. A color key is given on the right side, and gradations correspond to *p *values, bootstrap percentages, and number of conflicting bipartitions, left to the right respectively. The arrow locates the concatenated tree.

The results of the approximately unbiased (AU) test indicated that 39 out of 260 genes rejected the concatenated tree. The *p *value heatmap (Figure 5a) indicates that some gene trees showed the same or very similar histories, depicted by groups of topologies with a similar *p *value pattern (for example, topologies 1 to 47, and 48 to 65). On the other hand, a small group of genes rejected most topologies (that is, genes 230 to 260, read horizontally in Figure 5a), and at the same time, their trees were rejected by most of the genes (that is, topologies 230 to 260, read vertically in Figure 5a). Although different topologies were supported by various groups of genes, the majority of genes did not reject the concatenated tree and only a small subset of genes proposed significantly different trees. The analysis of bipartitions (Figure 5b) demonstrated that the vast majority of genes supported three distinct bipartitions, corresponding to the monophyly of *S. pyogenes*, *S. pneumoniae *and *S. thermophilus *(bipartitions 28, 29, and 30, respectively). Also generally supported were the monophyly of *S. agalactiae*, the monophyly of the group *S. pneumoniae *+ *S. suis*, and the monophyly of the group *S. agalactiae *+ *S. pyogenes *(bipartitions 27, 26 and 25, respectively). Several other bipartitions were only supported by some genes (for example, bipartition 19, corresponding to the grouping of *S. pneumoniae *with *S. thermophilus*), while others were only supported by one or a few genes (for example, bipartition 10 and 11). The well supported conflicting bipartitions figure (Figure 5c) is a summary of the *p *value heatmap (Figure 5a) and bipartition analyses (Figure 5b). A majority of the genes (around 150 out of 260) show no conflict with each other. Most of them support the monophyly of the different species and the lineage *S. pneumoniae *+ *S. suis*, and most of them do not reject the concatenated gene tree. Another set of genes showed some instances of conflict with the aforementioned set of 150, but most of them were in conflict with each other. They tend to support the same principal groups as the set of 150, with a few additional bipartitions that are conflicting. A final group of genes conflict with the first and the second group, as well as with each other, corresponding to genes that rejected most of the other gene trees in the AU test (Figure 5a) and that provide support for rare bipartitions; genes of this set have strongly incongruent histories with the other genes (for a detailed list, see Additional data file 4). The topologies used to test for positive selection were the concatenated gene tree for the genes that don't reject it, and individual gene trees for those loci that do reject the concatenated tree.

#### Within *S. agalactiae*

**Figure 6:**
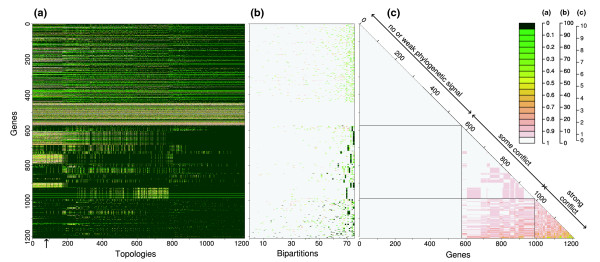
*S. agalactiae *recombination heatmaps. The layout is the same as Figure 5 but for the core-genome of *S. agalactiae*.

The concatenated gene tree was rejected by 750 genes of the core-genome of *S. agalactiae*. On the whole, most genes rejected most of the other gene trees (Figure 6a), although there were also some genes that did not reject the majority of gene trees. There were no commonly well supported bipartitions across the genes (Figure 6b). Around half of the genes provided either no, or only weak, bootstrap support for any bipartition (genes 1 to 560; Figure 6b), while the rest of the genes supported different sets of bipartitions. The most commonly supported groups of strains were 515+NEM316, A909+H36B, 515+NEM316+COH1, A909+CJB111+H36B, A909+CJB111+H36B, and 515+COH1 (bipartitions 75 to 70, respectively; Figure 6b). Additional, numerous bipartitions were supported by only one or a few genes. Because they possessed a too limited phylogenetic signal, around half of the genes (genes 1 to 560) showed no conflict with any of the other genes (Figure 6c). Although the AU test suggested that some of these genes have different histories, it is difficult to reach any definitive conclusions about the congruence of these gene histories since phylogenetic signal was so limited or absent (genes with no sequence divergence between strains).

The second half of the core-genome can be split into two groups. The first group contains genes that have some conflict with each other, and that tend to support the six bipartitions described earlier, plus three additional ones. The second group contained genes that were largely in conflict with each other, and with the preceding group. This latter group provided support for a number of rarely supported bipartitions. While the first group contained genes that had only partly incongruent histories (only a few bipartitions in conflict), genes of the last group had more incongruent gene histories (greater number of bipartitions in conflict). Given these results, and the ambiguity of defining which genes had the same history, we analyzed each gene with its own gene tree in the subsequent positive selection analyses.

#### Within *S. pyogenes*

**Figure 7:**
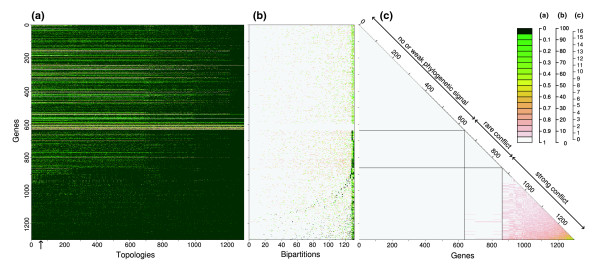
*S. pyogenes *recombination heatmaps. The layout is the same as Figure 5 but for the core-genome of *S. pyogenes*.

As for *S. agalactiae*, while a few genes rejected nothing, the majority of genes rejected the other gene trees (Figure 7a). Three bipartitions were generally supported, although not always, and with various bootstrap scores, corresponding with serotype groupings: MGAS5005+M1 GAS, MGAS315+SSI-1, and MGAS2096+MGAS9429 (bipartitions 131 to 129, respectively; Figure 7b). A total of 434 genes tended to also provide support for various unique bipartitions. Around half of the genes had weak or no phylogenetic signal, and, as a consequence, had no conflict with any other trees (Figure 7b). A set of around 200 genes, most of which supported the three bipartitions detailed above, tended not to conflict with each other, but occasionally with the final grouping of genes. This latter group was composed of the 434 genes mentioned above, which supported variously different bipartitions, and thus tended to be in conflict with each other. Overall, the *S. pyogenes *core-genome is composed of genes that are largely congruent for a portion of relatively recent history (that is, the serotype monophyly), while one-third of the core-genome appears to have strongly incongruent histories for older events. Because it appeared difficult to define which genes were likely to have the same history, we analyzed each gene with its own gene tree in the subsequent positive selection analyses.

#### Substitution analysis of recombination

The pairwise homoplasy index (PHI) approach suggested that around 20% of the genes were recombinant within the core-genome of *Streptococcus *and *S. pyogenes*, while within *S. agalactiae *only about 3% of the genes were recombinant (Table 2). Employing a more conservative approach that considers as recombinant only those genes found by three different substitution approaches (PHI, MaxChi and neighbor similarity score (NSS)), these proportions were reduced, but the relative differences between the data sets remained (Table 2). With the phylogenetic approach detailed above, numerous genes had weak phylogenetic signal, and several groups of genes were only partially incongruent; therefore, it can be difficult to define clearly which genes have different histories. It is, however, possible to adopt a conservative approach that considers as putative recombinants only those genes with strong phylogenetic incongruence (SPI), with most of the other genes. Nevertheless, only a small proportion of genes was identified by both PHI and SPI approaches as putative recombinants (Table 2), suggesting that each approach tends to identify different types of recombination event. We therefore propose that an estimate of the complete set of putative recombinants can best be considered as the set of genes identified by SPI plus the genes identified by all three substitution recombination methods (Table 2). This yields an estimate of 18% of the core-genome for *S. agalactiae *as putative recombinants, 19% for the genus *Streptococcus*, and 37% for *S. pyogenes*.

**Table 2:** Number of genes showing evidence of recombination

	1. SPI	2. PHI	3. PHI ∩ MaxChi ∩ NSS	1 ∩ 2	1 ∩ 3
Between species	26 (10.0%)	54 (20.8%)	35 (13.5%)	11 (4.2%)	53 (19.2%)
*S. pyogenes*	434 (33.5%)	284 (21.9%)	168 (12.9%)	186 (14.3%)	477 (36.8%)
*S. agalactiae*	222 (18.3%)	34 (2.8%)	7 (0.6%)	18 (1.5%)	223 (18.4%)

### Positive selection analysis

**Figure 8:**
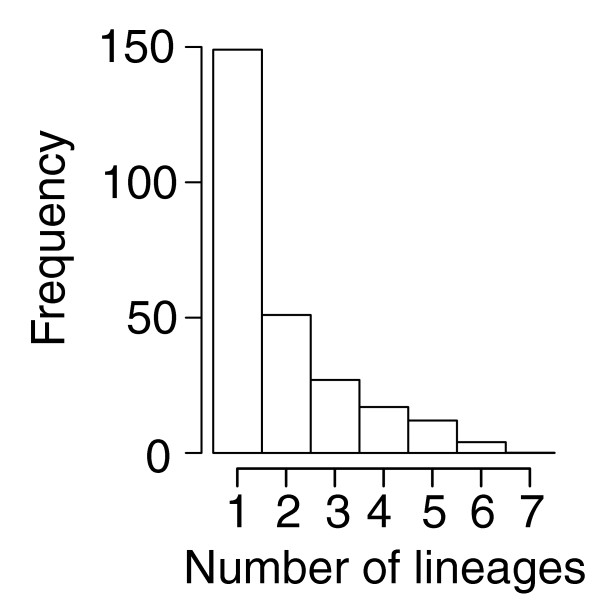
Frequency of positive selection. Numbers of genes showing evidence of positive selection in 1-7 lineages.

**Figure 9:**
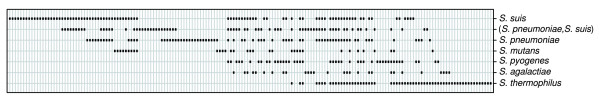
Positive selection occurrence per genes and lineages. A black dot indicates positive selection. The genes and lineages were ordered following a correspondence analysis.

The number of genes that showed evidence for positive selection was particularly high within the *Streptococcus *core-genome (between 10% and 40%; Table 3). The *S. pneumoniae *and *S. suis *lineages, and the ancestral lineage leading to these two species, exhibited the greatest proportion of the core-genome evolving under positive selection (28%, 34% and 32%, respectively; Table 3). Approximately one-third of the genes showed positive selection on only one lineage, and no gene was selected in all possible lineages (Figure 8). There were, however, many examples of genes selected on multiple lineages, including several genes selected on as many as 5 (12 genes) or 6 (4 genes) different lineages (Figures 8 and 9; see Additional data file 5 for a complete list of all genes and lineages under positive selection). A significant proportion of positively selected genes for *S. suis*, *S. pneumoniae*, and *S. thermophilus *was uniquely selected on each of these lineages (21%, 19%, and 24%, respectively), in contrast to that for *S. agalactiae*, *S. pyogenes*, and *S. mutans*, which had either no uniquely selected loci (*S. agalactiae*), or a very small proportion (Figure 9). Analysis of variance of genes under positive selection pressure supported a significant effect of both lineage and biochemical main role category (Table 4). *Post hoc *multiple comparisons showed that the main effect was due to two categories, 'DNA metabolism' and 'Transcription'. Less strongly supported, but still significant, was the interaction between lineages and main role categories (Table 4). This interaction appeared mainly due to an increase of genes under positive selection for loci involved in transcription, protein fate, protein synthesis and DNA metabolism for the *S. pneumoniae-S. suis *ancestral lineage and the *S. suis *lineage.

**Table 3:** Genes under positive selection

Data set	Lineage	n	PS	%
*Streptococcus*	*S. mutans*	260	33	12.69
	*S. pneumoniae*	260	73	28.08
	*S. suis*	260	89	34.23
	*S. thermophilus*	260	61	23.46
	*S. agalactiae*	260	28	10.77
	*S. pyogenes*	260	44	16.92
	(*S. pneumoniae*, *S. suis*)	221	71	32.13
*S. agalactiae*	COH1	1,212	7	0.58
	18RS21	1,212	0	0.00
	NEM316	1,212	1	0.08
	H36B	1,212	1	0.08
	A909	1,212	0	0.00
	2603V/R	1,212	1	0.08
	CJB111	1,212	1	0.08
	515	1,212	0	0.00
*S. pyogenes*	MGAS10270	1,297	7	0.54
	MGAS10394	1,297	3	0.23
	MGAS10750	1,297	1	0.08
	MGAS2096	1,297	1	0.08
	MGAS315	1,297	0	0.00
	MGAS5005	1,297	1	0.08
	MGAS6180	1,297	2	0.15
	MGAS8232	1,297	4	0.31
	MGAS9429	1,297	2	0.15
	M1 GAS	1,297	0	0.00
	SSI-1	1,297	0	0.00
	(MGAS9429, MGAS2096)	925	2	0.22
	(MGAS5005, M1 GAS)	978	4	0.41
	(SSI-1, MGAS315)	983	9	0.92
*S. thermophilus*	CNRZ1066	1,365	3	0.22
	LGM 18311	1,365	3	0.22
	LMD-9	1,365	14	1.03

PS, positive selection.

**Table 4:** Analysis of variance for the effect of the lineages and role categories

	Df	Sum Sq	Mean Sq	F value	*p *value
Lineage	6	2,954	492	23.9	<0.0001
Main role	10	1,086	109	5.27	<0.0001
Interaction	60	1,974	33	1.6	0.003
Residuals	1,699	35,005	21		

Df, degree of freedom.

In addition to identifying genes and lineages under positive selection, the branch-site test also identifies sites using a Bayes empirical Bayes approach [25]. For 91% of the genes under positive selection, specific sites were proposed (posterior probability >0.95). Interestingly, when a gene was independently selected on different lineages, the sites under positive selection were generally not the same across lineages, arguing for different selection pressure located at different sites. In contrast to the interspecific comparisons, positive selection was evident for only a few genes within the core-genome, across strains of the different *Streptococcus *species (Table 3, Additional data file 5), including a few lineages that showed slightly increased levels of positive selection relative to the rest. For *S. agalactiae *the exceptional lineage was COH1, for *S. pyogenes *the exceptional lineages were MGAS10270 and that leading to SSI-1/MGAS315, and for *S. thermophilus *it was LMD-9. A significant number of genes evolving under positive selection were also judged as putative recombinants (Table 5). This was particularly true for the *S. pyogenes *genome, where 78% of the genes under positive selection were putative recombinants. Approximately half of these genes were identified as recombinants by the substitution based recombination methods, and the other half by the phylogenetic approach.

**Table 5:** Total number of genes showing evidence of positive selection and recombination

Data set	PS	PS and R	PS and SPI	PS and IR
Between species	175	43 (25%)	20 (8%)	29 (11%)
*S*.*agalactiae*	10	4 (40%)	4 (40%)	0 (0%)
*S*.*pyogenes*	32	25 (78%)	21 (65%)	17 (53%)

PS, positive selection; R, recombination.

## Discussion

### Core-genome, pan-genome, and recombination

We estimate that the pan-genome of the genus *Streptococcus *probably exceeds at least three times the average genome size of a typical *Streptococcus *species. This huge variability in gene content between species is also evident in comparisons across strains of the same species. Our prediction for the *S. agalactiae *pan-genome is in general agreement with that of Tettelin *et al*. [22]. The marked difference in estimated pan-genome size for these two species may be a reflection of their habitat differences. The human oral-nasal mucosa is the primary habitat for *S. pyogenes*, whereas *S. agalactiae *was first identified as a bacteria linked to bovine mastitis, and later in humans, where it colonizes the lower gastrointestinal tract and vaginal epithelium of healthy adults. This apparent broader habitat range for *S. agalactiae*, and presumably, therefore, a greater available gene pool for lateral gene transfer, could explain the difference in pan-genome size of these two species.

The pronounced evolutionary flexibility of these bacterial genomes is further evident in the determinations of gene gain, loss and duplication on each of the respective lineages. Gene gain figures were generally higher for *S. agalactiae *than for *S. pyogenes*, despite the fact that branch lengths suggest the *S. pyogenes *lineages may be older, and is likely a consequence of the overall smaller pan-genome size for *S. pyogenes*. For some species, gene gain figures exceeded 20% of the total gene content for the organism. Our results in this regard are in general agreement with those of Hao and Golding [23], while also extending the estimates to additional taxa of *Streptococcus*, and lineages of *S. agalactiae *and *S. pyogenes*, and we would certainly concur with these authors that much of this gene gain likely reflects species specific adaptation. In our opinion, a plausible explanation of the discrepancy between gene gain and loss is that much of the pan-genome remains unsampled, and, therefore, we simply cannot detect many gene loss events, resulting in an underestimate of that category. In several genome reconstruction analyses, gene gain is penalized with respect to gene loss (for example, [24,26-28]). This procedure logically results in an increase of gene loss relative to gene gain. It has the direct consequence of increasing ancestral genome sizes, and reducing the importance of LGT events to the benefit of genome reduction processes. Nevertheless, this approach is questionable when one attempts to reconstruct genome composition, and in particular, for the case of *Streptococcus*. First, because the genome size is relatively stable in *Streptococcus*, the ancestral genome sizes were arguably of the same order. Second, the number of genome specific genes is so common that there is little reason to postulate that gene gain is less probable than gene loss. Therefore, at least in the case of *Streptococcus*, we question the validity of an approach that favors genome reduction processes over LGT. In that regard, the development of probabilistic models will be highly valuable to estimate rates of gene gain and loss (for example, [16,29]).

In contrast to the pan-genome, the core-genome size of the taxonomic groups included in our analysis are much better estimated. Within *Streptococcus*, we characterized several core-genomes, the composition of which depended on the taxonomic level considered. Our prediction of core-genome composition for *S. agalactiae *was in general agreement with Tettelin *et al*. [22] and Brochet *et al*. [30]. The slight differences in the absolute numbers between the three studies are due to differences in methodology used to define orthology [22], or the use of DNA microarray hybridization data [30]. At the genus level, the core-genome corresponded to 25% of a typical *Streptococcus *genome, while at the species level it represented around 60% of the genome. Earlier studies involving other groups of bacteria have suggested that such core-genomes may be relatively free of recombination [31-33]. If you consider the union of both substitution based methods and phylogenetic based methods we estimate that around 18% of the core genome of the genus *Streptococcus *is recombinant and as much as 35% of the genome of *S. pyogenes*. In addition to the fact that we are analyzing a different group of taxa, and thus levels of recombination might well be expected to be different, our results differ from these earlier estimates, also because of approach. We concur with the comments of Susko *et al*. [34] that attempts to evaluate phylogenetic congruence of core-genes need to involve comparisons with as many relevant topologies as possible, and not just that favored by the concatenated topology, some variants of it, or particular canonical markers such as the small subunit rRNA. In doing this, however, we think it is important to keep in mind that topologies may be rejected as being congruent, even though the genes may provide little phylogenetic signal, and, thus, only that proportion of the gene trees that is rejected based on strongly supported conflicting nodes should be regarded as incongruent. This was particularly evident in our analysis of *S. pyogenes*, where the vast majority of topologies reject one another despite the fact that at least half of these genes have little or no phylogenetic signal. Furthermore, our assessment of core genome recombination also differs from some of these earlier studies by the inclusion of substitution based approaches to recombination detection. There was little overlap in the loci identified as putative recombinants using both the phylogenetic and the substitution based approaches, suggesting they were identifying very different types of recombination. The substitution based approaches are likely to detect homologous recombination of smaller pieces of DNA that could be missed by a phylogenetic approach. The more restricted habitat distribution for *S. pyogenes *may also be an explanation for the elevated amounts of recombination in the core-genome of that species. A more reduced gene pool for possible recombination would not only result in a smaller pan-genome size (as suggested above for *S. pyogenes*), but it would also result in the propensity for more homologous recombination of core-genome components, at least partly because the relative proportion of conspecific donor pieces of DNA are likely to be greater.

### Positive selection in the core-genome

A logical surmise often made in studying pathogen evolution is that much of the host specific adaptation that a bacterial species exhibits will be associated with its species specific genes. Perhaps as a consequence of this common sense viewpoint, adaptation within the core genome has received much less attention. Our analysis reveals that during the diversification of the genus *Streptococcus *there has been significant amounts of positive selection pressure on core genome components and that this selection pressure has occurred disproportionately in certain lineages, and biochemical categories. Such an important positive selection signature within the core-genome of *Streptococcus *is perhaps somewhat surprising, as these predominately housekeeping genes might be expected to evolve under strong purifying selection. At the same time, we would argue that many of these genes are undoubtedly related to the colonization, persistence, survival, and propensity to cause disease in these organisms and thus are associated with the adaptive specifics of the bacteria.

Based on currently available phylogenies for the genus *Streptococcus *[35], there appears to be a rough correlation with the relative divergence of taxa and the level of positive selection detected in different lineages. For example, *S. pyogenes *and *S. agalactiae *are both members of the Pyogenes taxonomic group and there are fewer numbers of genes selected on these lineages than for taxa from different taxonomic groups. Several genomes also tend to resemble one another in relation to the genes that were positively selected, while others, such as *S. suis *and *S. thermophilus*, exhibit higher levels of specific adaptation. *S. suis *was also the species with the largest number of positively selected genes in its core-genome, relative to the other lineages, and the genome that had the greatest amount of gene gain and loss incurred since the separation of the *S. pneumoniae-S. suis *common ancestor. This suggests a lineage that has been under strong selection pressure, both with regard to acquiring new genes and with regard to the sequence characteristics of the core genome components. This selection pressure is undoubtedly correlated with particular characteristics of the host species, swine. Current phylogenies support *S. acidominimus *as the sister group to *S. suis *[35], a species associated with bovine vagina, the skin of calves, and raw milk, suggesting the possibility of host divergence in one or another (or possibly both) lineage, subsequent to their split from a common ancestor. *S. suis *is also known as an occasional zoonotic pathogen of humans, causing septicemia, meningitis, endocarditis [36], and, most recently, streptococcal toxic shock syndrome [15]. Our analysis suggests an apparent evolutionary flexibility of the *S. suis *genome that could perhaps be related to this propensity for host jumping.

In addition to the lineage effect in the interspecific selection analysis, there was also an effect of biochemical roles, with DNA metabolism and transcription significantly different from the majority of the other categories and correlated with higher incidence of positive selection. An excess of positive selection in the genes related to transcription is perhaps surprising, as these genes are generally well conserved and are known to be particularly recalcitrant to recombination. Furthermore, the *S. suis *and *S. pneumoniae-S. suis *ancestral lineages showed a disproportionate amount of positively selected genes for several biochemical categories. Thus, the extent of adaptive molecular evolution varies across lineages and gene roles, undoubtedly reflecting the habitat heterogeneity of the genus *Streptococcus*. Our results also reveal that positive selection was partly linked to recombination. Recombination can create artifactual positive selection results [37], particularly for genes showing evidence of intragenic recombination. This is less probable for genes identified as recombinant using the phylogenetic recombination detection procedure, because these loci were analyzed for positive selection with their own phylogenies, thereby taking into account possible LGT events. It is possible that many LGT loci could also be positively selected because of strong selective forces that might be expected to act on newly acquired genes. Marri *et al*. [16] found that many species specific genes within the *Streptococcus *genus showed evidence of adaptive evolution, and concluded that LGT played an important adaptive role.

Although a good deal of positive selection pressure was evident in the analysis of different lineages within the genus *Streptococcus*, there was much less evidence for positive selection between the different lineages of *S. agalactiae*, *S. pyogenes*, and *S. thermophilus*. Nevertheless, these absolute numbers of genes under positive selection are not corrected for depth of ancestry. In other words, they do not reflect rates of adaptative mutations for specific lineages; instead, they represent the core-genome fraction that has participated in the adaptation of a specific taxon. There were, however, several lineages in each of these species that had slightly elevated levels of selection relative to the rest. In *S. pyogenes*, for example, the lineage leading to the M3 serotype had nine genes under positive selection, while the majority of other lineages had two or less (see Additional data file 5 for a complete description of these genes). Compared to other M types, serotype M3 strains cause more cases of invasive disease, such as necrotizing fasciitis, bacteremia, and streptococcal toxic shock syndrome [38-42], a higher rate of lethal infections [41-43], and exhibit occasional epidemic tendency [38]. The nine genes we identified as positively selected included loci implicated as virulence determinants in other species of *Streptococcus*, such as *adenylosuccinate lyase*, a homotetramer that catalyzes two discrete reactions in the *de novo *synthesis of purines, and has recently been implicated as a virulence factor for infective endocarditis, a serious endovascular infection caused by *Streptococcus sanguinis *[44], as well as genes involved in cell envelope, such as *UDP-N-acetylmuramoylalanyl-D-glutamyl-2,6-diaminopimelate-D-alanyl-D-alanyl ligase*, demonstrated to be integral to peptidoglycan biosynthesis and cell growth [45]. In addition to the evaluation of genes unique to M3 strains (for example, [46]), we suggest that these nine core-genome loci uniquely under positive selection pressure in this M3 lineage should be considered as putatively important in the unique pathogenic features of M3 strains.

In the case of *S. agalactiae*, the lineage that stood out from the rest with regard to levels of positive selection pressure was COH1, which is serotype III, ST17, significantly associated with neonatal invasive disease [47], and is hypothesized to have recently arisen from a bovine ancestor [48]. The seven genes selected on this lineage include a number of loci either already implicated in virulence in other bacteria, or for which there is some reason to suspect them as candidate virulence loci (see Additional data file 5 for a full description of these genes). For example, *adenylosuccinate lyase*, discussed above with regard to *S. pyogenes*, was also positively selected in this lineage. *Phosphate acetyltransferase *was uniquely selected along this COH1 lineage, and has recently been implicated in virulence in *Salmonella enterica *[49]. Also selected were a protein involved in the cell envelope, as well as two different ABC transporters. The cell envelope is a key overall component of virulence in *S. agalactiae *[50]. ABC transporters have been known for some time to be efflux mechanisms of drug resistance, although such efflux pumps are now also known to have physiological roles, conferring resistance to natural substances produced by the host, as well as possible roles in pathogenicity [51]. Perhaps the proposed recent shift in host preference from bovine to human for this lineage [48] is facilitated by molecular adaptation of ABC transporters that confer resistance to natural substances of the new host.

## Conclusion

The research presented here employs a comparative genomics approach to define the core-genome component of the genus *Streptococcus*, as well as that of *S. agalactiae*, *S. pyogenes*, and *S. thermophilus*. We then assess levels of recombination and positive selection pressure in this core-genome for each of these taxonomic groups. Concomitant with these assessments of core-genome were estimates of the pan-genome size of each of these groups, and levels of gene gain, loss and duplication on each of the lineages.

The pan-genome size of *S. pyogenes *appears to be quite well estimated with the 11 sequences currently available, and is approximately 2,500 genes. The pan-genome size of *S. agalactiae *is less well estimated with available sequence data and is in excess of 2,800 genes. Similarly, the pan-genome size of the genus *Streptococcus *is not accurately estimated with the 26 genomes analyzed here, and is in excess of 5,300 genes. We suggest that the broader habitat range for *S. agalactiae *may provide a greater available gene pool for lateral gene transfer, and could explain the difference in pan-genome size of *S. agalactiae *and *S. pyogenes*.

The core-genome components of each of these taxonomic groups is much better represented, and contrary to some earlier studies involving other groups of bacteria, which have suggested that such core-genomes may be relatively free of recombination, we estimate that around 18% of the core-genome of the genus *Streptococcus *is recombinant and as much as 35% of the genome of *S. pyogenes*. An explanation for the greater amount of recombination in *S. pyogenes *may be related to the more restricted habitat distribution for *S. pyogenes*, which would result in the propensity for more homologous recombination of core-genome components because the relative proportion of conspecific donor pieces of DNA is likely to be greater. Positive selection across the core-genome was particularly evident in the analysis of the different species within the genus *Streptococcus*, and it occurred disproportionately in certain lineages, as well as biochemical categories. *S. suis *was the lineage that showed the greatest positive selection pressure, the largest number of loci uniquely selected, and the lineage that had the greatest amount of gene gain and loss. In addition to the lineage effect in the interspecific selection analysis, there was also an effect of biochemical role, with genes related to DNA metabolism and transcription showing a significantly higher number of genes under positive selection. Contrary to the interspecific analysis, the selection analysis on individual species supported much less evidence for positive selection, but suggested there were particular lineages in each species that had experienced more core-genome selection pressure than the others. In the case of *S. pyogenes *this was the lineage leading to the M3 serotype, and we suggest that the nine genes identified as positively selected should be considered as putatively important in the unique pathogenic features of M3 strains. In the case of *S. agalactiae *the lineage with the disproportionate selection pressure was COH1, which is known to be significantly associated with neonatal invasive disease, and is hypothesized to have recently arisen from a bovine ancestor. We suggest that this proposed recent host jump from bovine to human for this lineage could be the explanation for the greater amount of selection pressure observed in this genome.

Overall, this study indicates that there has been considerable recombination and positive selection pressure in the diversification of the *Streptococcus *core-genome, particularly at the interspecific level. Positive selection seems to be of principal importance in species differentiation and adaptation to new hosts, while it plays a less important role during strain evolution, where the process may be too slow to facilitate rapid strain adaptation. On the other hand, the process of recombination, through either LGT or homologous intragenic recombination, involving both the core-genome and the pan-genome, appeared to be of main importance at a variety of evolutionary time scales. It seems likely that recombination is a more efficient means of change, ultimately making it the more universal process of *Streptococcus *adaptation. Although the cause-effect explanations are not necessarily clear for many of the genes that we identify as positively selected, it is nonetheless important to realize that positive selection of these genes indicates such loci have important functions, which in many instances may be integral to the unique adaptive features of each lineage. Several recent studies have harnessed the power of modern molecular selection analyses to direct functional experimentation based on the resulting molecular evolutionary hypotheses (see for example, [52-54]). It is our hope and intention that the identification and cataloging of these loci (Additional data file 5) for this and other groups of bacteria will serve as an evolutionary shortcut for others to design laboratory mutation experiments to assess the specific functional significance of these genes.

## Materials and methods

### Ortholog retrieval

Twenty six genomes of *Streptococcus *were downloaded from GenBank (Table 1), representing six different species. Coding sequences were extracted from GenBank files, and orthologs were determined using OrthoMCL [43]. This program first makes an all-against-all BLASTp, and then defines putative pairs of orthologs or recent paralogs based on reciprocal BLAST. Recent paralogs are identified as genes within the same genome that are reciprocally more similar to each other than any sequence from another genome. OrthoMCL then converts the reciprocal BLAST *p *values to a normalized similarity matrix that is analyzed by a Markov Cluster algorithm (MCL) [55]. In return, the MCL yields a set of clusters, with each cluster containing a set of orthologs and/or recent paralogs. OrthoMCL was run with a BLAST E-value cut-off of 1e-5, and an inflation parameter of 1.5. We used the OrthoMCL output to construct a table describing genome gene content (Additional data file 1). Genes that were not included in a cluster were considered taxon specific genes only if they were at least 50 amino acids long and had no BLAST hit with any other protein (E-value ≤ 1e-10). Preliminary analysis indicated that many truncated proteins found at the ends of contigs of the incomplete genomes, although exhibiting clear evidence of homology, were not included in any cluster because they had weak or no reciprocal BLAST hit. This table was used to plot venn diagrams with R 2.2.1 [56] and to construct four core-genome data-sets corresponding to the following taxa: genus *Streptococcus*, *S. agalactiae*, *S. pyogenes*, and *S. thermophilus*.

Gene loss, acquisition, and duplication were determined on all branches of trees involving these taxa using the parsimony criterion with the DelTran option, implemented in Paup 4.0b10 [57]. Ancestral gene state reconstruction was run with two different step-matrices: the first one assesses gene gain, loss and duplication as being equally likely (for example, [23]), while the second penalizes gene gain by assuming a double cost compared to loss and duplication (for example, [24]). The gene content table was also used to perform gene accumulation curves using R, which describe the number of new genes and genes in common, with the addition of new comparative genomes. The procedure was repeated 100 times by randomly modifying genome insertion order to obtain means and standard errors.

### Alignments

Orthologs were first aligned at the DNA level with ClustalW 1.82 [58]. To ensure homology, alignments that contained less than 35% conserved sites for the *Streptococcus *genus data set, and 50% for the *Streptococcus *species data sets, were discarded. A preliminary analysis of the data revealed that many sequences identified as under positive selection contained frameshifts that disrupted the reading frame (a single insertion or deletion that modified the reading frame), resulting in high non-synonymous substitution rates. Unfortunately, it is not possible to accurately discriminate sequencing errors from actual insertions or deletions; however, most of these frameshifts were found within the unclosed genomes and appeared at the beginning or end of the contigs, where sequencing errors are more probable. As described by Perrodou *et al*. [59], most of these frameshifts are probably sequencing errors, although it is possible that some are not [60]. We chose the conservative approach of removing all codons appearing before or after the frameshift when located at the beginning or end of the coding sequence, respectively. For that purpose, Perl scripts were developed to find frameshifts on the DNA alignments, and the sequences were edited manually. A second alignment step was then used to refine all alignments by translating sequences to amino acids, aligning them with ClustalW, and then back-translating to DNA, using the script transAlign [61]. Finally, amino acid alignments showing a low percentage of conserved sites were manually inspected, and removed if the alignment was ambiguous.

### Recombination detection

Both phylogenetic and substitution pattern methods were use to detect recombination events. The phylogenetic methods rely on the examination of phylogenetic congruence among genes (for example, [62,63]). If a gene has been laterally transferred, the phylogenetic relationships depicted by this gene will be different from the species tree. We first applied the method suggested by Susko *et al*. [34], which tests for the rejection of a set of topologies by a set of orthologous genes using the AU test [64]. When possible (that is, the number of taxa ≤ 7), the trees tested are all the possible unrooted trees (for example, [65]). When a gene rejects a tree that is supported by the majority of the other genes, this gene is considered to have been laterally transferred. We applied this approach to our data sets (except for *S. thermophilus*, which contains only three taxa), by using as tested topologies the individual gene trees obtained by phyML (general time reversible (GTR) +Γ4+I model of evolution with a BIONJ starting tree) [66], with the addition of the tree obtained with Paup (GTR+Γ4+I model of evolution, neighbor-joining (NJ) starting tree, and a tree-bisection-reconnection (TBR) branch-swapping algorithm) reconstructed from the concatenation of all genes. The site likelihood of each tree was than computed by the program baseml (PAML package) [67] using a GTR+Γ4 model of evolution. The AU test was then applied using Consel [68].

As suggested by Susko *et al*. [34], results (*p *values for the rejection of each tree) were plotted using heatmaps obtained with R. This approach has the disadvantage of been developed with a test (the AU test) that assesses the rejection of a tree by a gene and not for its acceptance [34]. As a result it is not possible to say if a tree is not rejected because it is not significantly different or because it is simply unresolved. This sort of situation is particularly expected with weakly divergent alignments, and at the opposite spectrum, with saturated alignments. We thus developed and performed a second set of analyses to complement the Susko approach and intended to quantify the amount of supported and incongruent phylogenetic signal between two gene trees. This approach relies on the discovery of well supported conflicting bipartitions (that is, branches that can not be observed in the same tree), as measured by non-parametric bootstrap analysis [69], thus revealing incongruence between gene histories. Support for each bipartition was obtained by bootstrapping a maximum likelihood (ML) tree search using Paup (GTR+Γ4+I model of evolution). Custom-made scripts were then used to find and count well supported (≥90% bootstrap support) conflicting bipartitions between gene trees. Additional to phylogenetic recombination detection, we employed methods specifically developed to detect homologous intragenic recombination. We used the compatibility approach between site histories, based on the pairwise homoplasy index (PHI), developed by Bruen *et al*. [70] and implemented within the program PhiPack [71]. Bruen *et al*. have suggested PHI is a more robust and sensitive method than many of the earlier approaches. Additional to the PHI statistic, for comparative purposes, we computed MaxChi [72] and NSS [73]*p *values for recombination using PhiPack (employing 1,000 permutations).

### Positive selection analysis

We employed the branch-site test of Yang and Nielsen [20,74] implemented in the program Codeml of the package PAML [67] to assess positive selection at particular sites and lineages. Briefly, the likelihood of a model that does not allow positive selection is compared to one allowing positive selection on some specified lineages. The model allowing positive selection is tested using a likelihood ratio test (LRT) that is compared to a χ^2 ^statistic with two degrees of freedom. Likelihoods were estimated on the genes or species trees. For the *Streptococcus *data set each lineage leading to the six different species was tested. For the species analyses each of the lineages corresponding with the different strains was tested, as well as several internal branches supported by the majority of genes. Finally, *p *values were corrected for multiple hypothesis testing using the Benjamini and Yekutieli method [75]. The effect of lineages, and genes' TIGR main role categories, and their interaction were tested using an analysis of variance on the LRT, using R. Role categories containing less than ten genes were merged. If F-statistic was significant, Tukey's 'honest significant difference' multi-comparison method was used to discriminate lineages and role categories associated with different LRTs.

## Additional data files

The following data are available with the online version of this paper. Additional data file 1 is a gene content table that describes the presence and absence of gene clusters per genome. Additional data file 2 is a CSV text file listing the composition of the clusters, with links to GenBank protein accession numbers. Additional data file 3 contains several tables detailing the gene gain, loss and duplication parsimony reconstruction for the *Streptococcus*, *S. pyogenes *and *S. agalactiae *pan-genomes. Results are presented for equal weights as well as following a model that penalized gene gain with respect to gene loss. Additional data file 4 is a table listing the genes showing evidence of recombination and positive selection. Additional data file 5 is a table listing the genes and lineages under positive selection for the four analyzed core-genomes, with gene annotation from NCBI and TIGR.

## Supplementary Material

Additional data file 1 Gene content table describing the presence and absence of gene clusters per genome Click here for file

Additional data file 2 Composition of the clusters, with links to GenBank protein accession numbers Click here for file

Additional data file 3 Results are presented for equal weights as well as following a model that penalized gene gain with respect to gene loss Click here for file

Additional data file 4 Genes showing evidence of recombination and positive selection Click here for file

Additional data file 5 Genes and lineages under positive selection for the four analyzed core-genomes, with gene annotation from NCBI and TIGR Click here for file
